# The Cytokinin Status of the Epidermis Regulates Aspects of Vegetative and Reproductive Development in *Arabidopsis thaliana*

**DOI:** 10.3389/fpls.2021.613488

**Published:** 2021-02-23

**Authors:** Sören Werner, Isabel Bartrina, Ondřej Novák, Miroslav Strnad, Tomáš Werner, Thomas Schmülling

**Affiliations:** ^1^Institute of Biology/Applied Genetics, Dahlem Centre of Plant Sciences (DCPS), Freie Universität Berlin, Berlin, Germany; ^2^Institute of Biology, NAWI Graz, University of Graz, Graz, Austria; ^3^Laboratory of Growth Regulators, Faculty of Science, Palacký University & Institute of Experimental Botany, The Czech Academy of Sciences, Olomouc, Czechia

**Keywords:** cytokinin, epidermis, *Arabidopsis*, shoot growth, seed yield, developmental transitions

## Abstract

The epidermal cell layer of plants has important functions in regulating plant growth and development. We have studied the impact of an altered epidermal cytokinin metabolism on *Arabidopsis* shoot development. Increased epidermal cytokinin synthesis or breakdown was achieved through expression of the cytokinin synthesis gene *LOG4* and the cytokinin-degrading *CKX1* gene, respectively, under the control of the epidermis-specific *AtML1* promoter. During vegetative growth, increased epidermal cytokinin production caused an increased size of the shoot apical meristem and promoted earlier flowering. Leaves became larger and the shoots showed an earlier juvenile-to-adult transition. An increased cytokinin breakdown had the opposite effect on these phenotypic traits indicating that epidermal cytokinin metabolism can be a factor regulating these aspects of shoot development. The phenotypic consequences of abbreviated cytokinin signaling in the epidermis achieved through expression of the ARR1-SRDX repressor were generally milder or even absent indicating that the epidermal cytokinin acts, at least in part, cell non-autonomously. Enhanced epidermal cytokinin synthesis delayed cell differentiation during leaf development leading to an increased cell proliferation and leaf growth. Genetic analysis showed that this cytokinin activity was mediated mainly by the AHK3 receptor and the transcription factor ARR1. We also demonstrate that epidermal cytokinin promotes leaf growth in a largely cell-autonomous fashion. Increased cytokinin synthesis in the outer layer of reproductive tissues and in the placenta enhanced ovule formation by the placenta and caused the formation of larger siliques. This led to a higher number of seeds in larger pods resulting in an increased seed yield per plant. Collectively, the results provide evidence that the cytokinin metabolism in the epidermis is a relevant parameter determining vegetative and reproductive plant growth and development.

## Introduction

The communication between different cell types is essential for the coordination of growth processes in multicellular organisms (Savaldi-Goldstein and Chory, 2008). In plants, the cells originate from meristems. Meristematic cells are arranged in an organized layered pattern which are maintained by the controlled orientation of cell divisions within layers. All above-ground organs originate from three clonally distinct cell layers in the shoot apical meristem (SAM): the cells of the single-row L1 and L2 layers only divide anticlinally and form the epidermis and subepidermal tissues, respectively, while the multidirectionally dividing cells of the L3 layer produce the SAM corpus and the different cell types of the ground and vasculature tissues (Ha et al., 2010). Coordination of cell division and cell expansion between these different cell layers is important and is achieved by exchange of phytohormones and other endogenous factors as well as external signals (Ingram, 2004).

So far, little is known about the contribution of the three cell layers of the SAM and the autonomous and non-autonomous factors that they produce to regulate development and growth of shoot lateral organs. It is clear however, that the outer cell layer (epidermis) forms not only a protective barrier to the environment (Yeats and Rose, 2013) but that it plays also a role in growth control (Czesnick and Lenhard, 2015). Analysis of periclinal chimeras of tobacco indicated that cell proliferation in the epidermis affects the proliferative activity of the subepidermal tissues (Marcotrigiano, 2010). Several studies have shown that cells in the epidermis both promote and restrict shoot growth by sending signals to the inner layers (Savaldi-Goldstein and Chory, 2008). For example, transgenic expression of a brassinosteroid synthesis gene specifically in the epidermis was sufficient to rescue the dwarf phenotype caused by mutation of the respective gene, which is expressed also in inner tissues. This indicated that the epidermis produces a signal promoting cell division and growth in inner tissues (Savaldi-Goldstein et al., 2007). In contrast, proliferation in inner leaf tissues was repressed by an epidermal signal mediated by very long chain fatty acids (Nobusawa et al., 2013). The main site of ethylene action in controlling leaf growth in *Arabidopsis* is the epidermis (Vaseva et al., 2018). Vaseva et al. (2018) have also demonstrated that ethylene-mediated cell expansion of the epidermis is rate-limiting for leaf growth. Much less is known about the role of the epidermal cell layer in other shoot organs such as the reproductive structures.

Another factor regulating plant growth and morphogenesis is the hormone cytokinin (CK) (Hwang et al., 2012; Kieber and Schaller, 2018; Werner and Schmülling, 2009). In the shoot, CK stimulates mitotic cell divisions and retards cell differentiation. Accordingly, plants with a lowered CK content formed smaller SAMs and displayed a reduced shoot growth (Werner et al., 2001, 2003). On the other hand, plants with an increased CK content or signaling showed opposite effects (Bartrina et al., 2011, 2017; Kiba et al., 2013). During reproductive development of *Arabidopsis*, CK has a profound influence on several processes. It regulates the inflorescence meristem activity and thus the number of flowers and siliques (Bartrina et al., 2011), the size of the gynoecia, and it acts as a positional cue during ovule formation (Bartrina et al., 2011; Zúñiga-Mayo et al., 2018, 2019), determines the duration of flowering (Bartrina et al., 2017) and is involved in seed size regulation (Werner et al., 2003; Riefler et al., 2006; Li et al., 2013). *Arabidopsis* plants defective in certain CK degradation genes have a higher seed yield (Bartrina et al., 2011).

The CK metabolism and signaling genes of *Arabidopsis* are known. Isopentenyltransferases (IPTs) and cytochrome P450 monooxygenases (CYP735A1 and CYP735A2) catalyze the formation of *N*^6^-isopentenyladenine (iP) and *trans-*zeatin (*t*Z) ribotides (Kakimoto, 2001; Takei et al., 2001, 2004; Miyawaki et al., 2006). The corresponding bioactive free bases are released by CK phosphoribohydrolases, belonging to the LONELY GUY (LOG) family (Kurakawa et al., 2007; Kuroha et al., 2009). The levels of active CKs are reduced either via degradation by CK oxidase/dehydrogenases (CKXs) or inactivation by glycoconjugation (Hou et al., 2004; Werner et al., 2006).

The CK signal transduction pathway is a His-Asp phosphorelay constituting a plant two-component signaling system (Kieber and Schaller, 2014). The *Arabidopsis* genome encodes three membrane-bound CK receptors ARABIDOPSIS HISTIDINE KINASE 2 (AHK2), AHK3, and CYTOKININ RESPONSE 1 (CRE1)/AHK4 (Inoue et al., 2001; Suzuki et al., 2001; Yamada et al., 2001). From the receptors, the signal is transmitted via histidine phosphotransfer proteins (AHPs) to transcription factors, type-B response regulators (ARRs), which regulate CK target genes (Sakai et al., 2000, 2001; Hwang and Sheen, 2001; Tanaka et al., 2004; To et al., 2004; Brenner et al., 2012; Bhargava et al., 2013; Zubo et al., 2017). Artificial repression of CK signaling can be achieved by translational fusion of a modified amino acid motif from class II ethylene response factors (ERFs) named SRDX (Ohta et al., 2001; Hiratsu et al., 2003) to type-B ARRs (Heyl et al., 2008).

Not much is known about the putative involvement of epidermal CK metabolism and signaling in growth regulation. In the SAM, CK synthesized in the L1 layer is predicted to form a gradient over several cell layers and position the organizing center, suggesting that it can act as a paracrine signal (Chickarmane et al., 2012; Gruel et al., 2016). Consistently, the epidermal cell layer expresses several CK metabolism and signaling genes (Yadav et al., 2014). CK reporter and marker gene analysis suggested that the entire leaf epidermis has the potential to sense CK and modify CK levels (Vatén et al., 2018). CK regulates the formation of stomata (Vatén et al., 2018) and promotes stomatal closure in defense to pathogens (Arnaud et al., 2017) showing that it has specific functions in the leaf epidermis. It is not known whether epidermal CK has specific functions in reproductive tissues.

We were interested to explore whether an altered CK metabolism or signaling in the epidermis would have an impact on growth and development of the plant shoot. To this end we have generated transgenic *Arabidopsis* plants expressing the *LOG4* or *CKX1* genes under control of an epidermis-specific promoter. In addition, we have expressed the CK repressor gene *ARR1*-*SRDX* in the epidermis in order to abbreviate CK output in a cell-autonomous fashion. The results showed that perturbations of CK metabolism in the epidermis impact shoot development consistent with the possibility that the epidermis is a source or sink of the hormone. Abbreviating CK signaling in the epidermis revealed a contribution of epidermal CK to leaf size regulation, presumably through regulating cell division during early leaf development.

## Results

### Targeted Expression of *LOG4*, *CKX1*, and *ARR1-SRDX* in the Epidermis Alters Shoot Development

**FIGURE 1:**
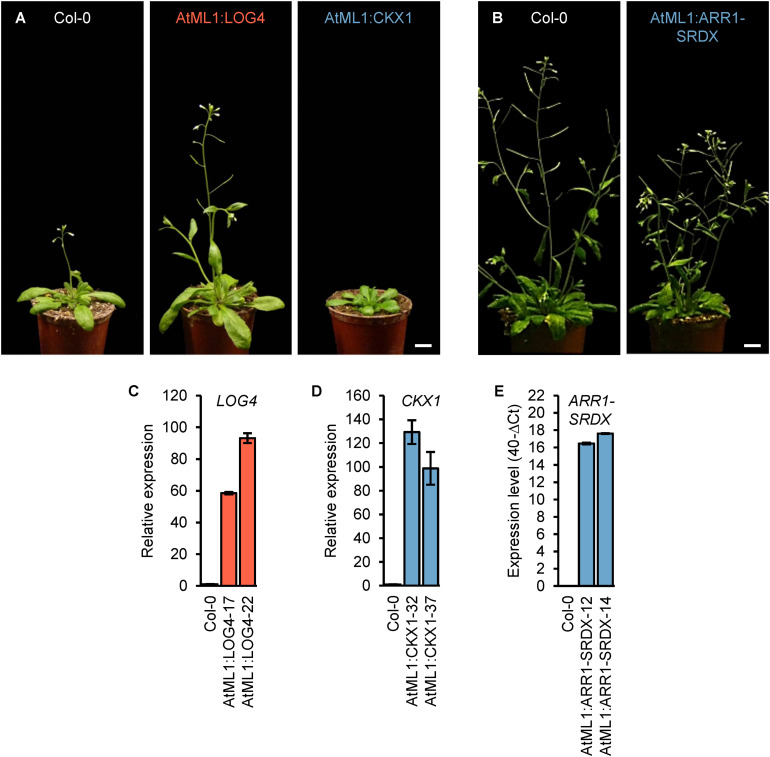
Expression of *ARR1-SRDX*, *CKX1*, and *LOG4* under the control of the *AtML1* promoter influences the development of *Arabidopsis* plants. **(A)** Phenotypes of transgenic AtML1:CKX1 and AtML1:LOG4 plants compared to wild-type plants grown under LD conditions (31 DAG); scale bar = 1 cm. **(B)** Phenotype of transgenic AtML1:ARR1-SRDX plants compared to the wild type grown under LD conditions (36 DAG); scale bar = 1 cm. **(C–E)** Expression level of *LOG4* **(C)**, *CKX1* **(D)**, and *ARR1-SRDX* **(E)** in shoots of 13-day-old plants grown under LD conditions. Transcript levels were determined by qRT-PCR, data were normalized to *PP2AA2* and *TAFII15* **(C,D)** or *PP2AA2* **(E)**, values are mean ± SEM, *n* = 4.

In order to investigate a possible role of epidermal CK to regulate shoot organ growth, we expressed *LOG4*, *CKX1*, and *ARR1-SRDX* in the epidermis under the control of the *AtML1* promoter. This promoter provides a highly specific expression limited to the epidermis of the growing shoot (Sessions et al., 1999) and has been used in several studies (Grandjean et al., 2004; Savaldi-Goldstein et al., 2007; Takada and Jürgens, 2007; Takada et al., 2013). The epidermal specificity of the cloned promoter fragment was verified in *AtML1*:*GUS* transgenic lines (Supplementary Figure S1). We generated independent transgenic lines (*n* > 17) expressing *AtML1*:*LOG4*, *AtML1*:*CKX1*, and *AtML1*:*ARR1-SRDX* constructs. Two representative lines per construct expressing the transgene and displaying typical phenotypic changes (Figure 1) were selected for detailed analysis.

**FIGURE 2:**
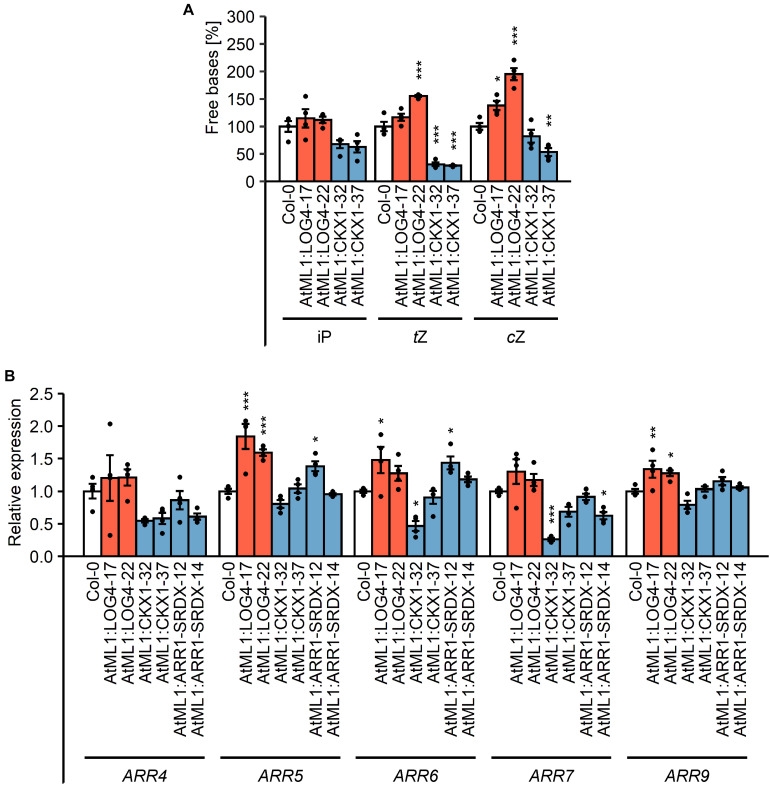
Impact of the expression of *AtML1:LOG4* and *AtML1:CKX1* on the CK status. **(A)** Amount of free CK bases in the transgenic lines relative to the wild type. Twenty milligrams of shoot material of 7-day-old seedlings grown under LD conditions were harvested and pooled for every biological replicate. Values are mean ± SEM (*n* = 4). Asterisks indicate significant differences compared to the wild type, as calculated by Kruskal–Wallis test, *post hoc* Dunn’s test (iP) or one-way ANOVA, *post hoc* Dunnett’s test (*t*Z, *c*Z) (*p < 0.05; **p < 0.01; ***p < 0.001). iP, *N*^6^-isopentenyl adenine; *t*Z, *trans*-zeatin; *c*Z, *cis*-zeatin; for a complete list of metabolites see Supplementary Table S1. **(B)** Transcript levels of A-type *ARR* genes in shoot material of 13-day-old LD-grown seedlings. Transcript levels were determined by qRT-PCR. Data were normalized to *PP2AA2* and *TAFII15.* Values are mean ± SEM (*n* = 4). Statistical analysis was conducted using one-way ANOVA, *post hoc* Dunnett’s test (*p < 0.05; **p < 0.01; ***p < 0.001).

In order to test how the epidermis-specific expression of *LOG4* and *CKX1* affects the CK concentration in the plants, CK metabolites were measured (Figure 2A, Table 1, and Supplementary Table S1). In AtML1:LOG4 seedlings, the total CK content was significantly increased by up to 17% in comparison to the wild type (Table 1). The concentrations of biologically active free bases were increased, in particular of *t*Z and *c*Z (Figure 2A and Supplementary Table S1). In contrast, *AtML1*:*CKX1* expression reduced the total CK content to about one-third compared to the wild type (Table 1) affecting most of the analyzed metabolites, including active CK bases (Figure 2A and Supplementary Table S1). Transcript levels of five selected CK-responsive A-type *ARR* genes, *ARR4*, *ARR5*, *ARR6*, *ARR7*, and *ARR9*, were tested. The steady state mRNA levels of these *ARR* genes were increased in AtML1:LOG4 and reduced in AtML1:CKX1 plants (Figure 2B). This suggested that the altered CK levels caused corresponding changes of expression of genes controlled by the hormone. In AtML1:ARR1-SRDX plants, *ARR4* and *ARR7* showed a downregulation, indicating repression of CK signaling (Figure 2B).

**TABLE 1:** Cytokinin concentration in Col-0, AtML1:LOG4, and AtML1:CKX1 plants.

Genotype	iP	*t*Z	*c*Z	Total
	metabolites	metabolites	metabolites	CKs
Col-0	36.97 ± 2.22	92.44 ± 6.43	7.35 ± 0.49	136.77 ± 8.59
AtML1:LOG4-17	35.80 ± 2.08	98.54 ± 4.02	6.56 ± 0.20	140.90 ± 6.01
AtML1:LOG4-22	41.85 ± 1.67	111.27 ± 2.15**	6.77 ± 0.15	159.89 ± 3.71*
AtML1:CKX1-32	14.61 ± 0.84***	22.67 ± 1.82***	2.90 ± 0.15**	40.18 ± 2.77***
AtML1:CKX1-37	15.61 ± 0.70***	24.51 ± 1.29***	3.31 ± 0.14*	43.42 ± 2.00***

Twenty milligrams of shoot material of 7-day-old seedlings grown under LD conditions were harvested and pooled for every biological replicate. Values are pmol g FW ± SEM (*n* = 4). Cytokinin concentrations that are significantly different from the wild type are printed in bold. Asterisks indicate significant differences compared to the wild type, as calculated by one-way ANOVA, *post hoc* Dunnett’s test (iP, *t*Z, total CKs) or Kruskal–Wallis test, *post hoc* Dunn’s test (*c*Z) (*p < 0.05; **p < 0.01; ***p < 0.001). iP, *N*^6^-isopentenyladenine; *t*Z, *trans*-zeatin; *c*Z, *cis*-zeatin; for a complete list of metabolites see Supplementary Table S1.

Phenotypic analysis of the generated transgenic lines revealed a number of quantitative changes during vegetative and reproductive shoot growth. The most obvious changes were a larger rosette and early flowering in AtML1:LOG4 lines and a diminished rosette growth and retarded flowering in AtML1:CKX1 lines as compared to wild type (Figure 1A). The shoot growth of AtML1:ARR1-SRDX plants was reduced, however, the changes were less pronounced than in AtML1:CKX1 (Figure 1B; see further below).

### Altered Epidermal CK Metabolism Influences the Size and Activity of the Shoot Apical Meristem

**FIGURE 3:**
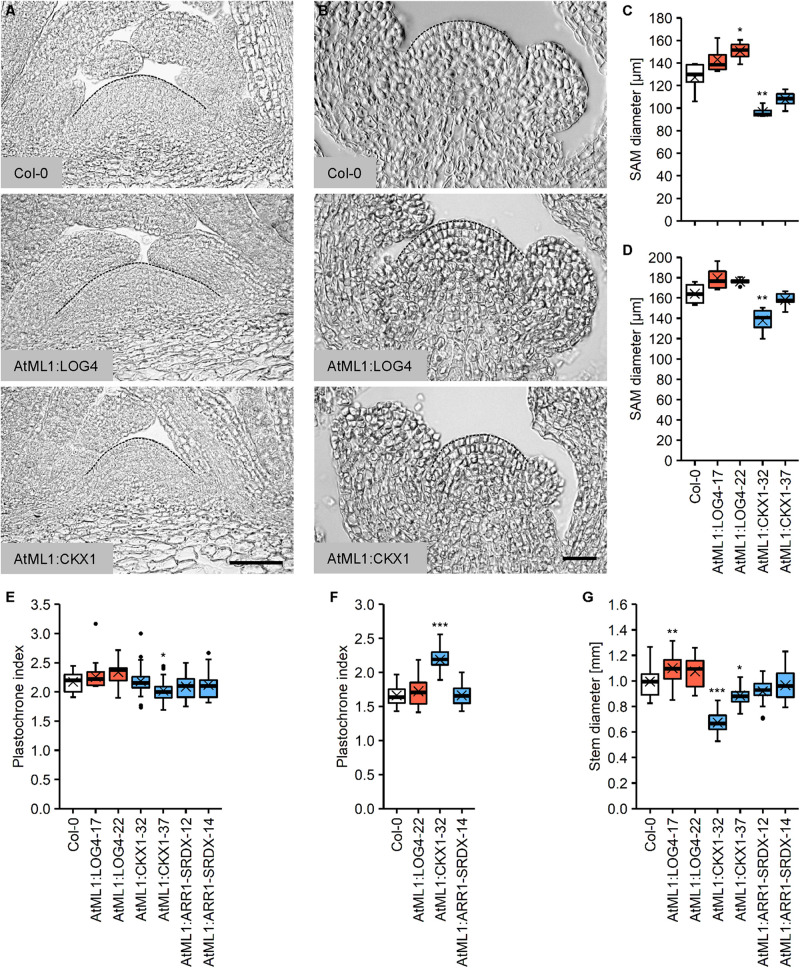
Altered epidermal cytokinin metabolism in AtML1:LOG4 and AtML1:CKX1 influences the size of the shoot apical meristem and the thickness of the stem. **(A)** Median longitudinal sections of the vegetative SAMs of 30-day-old SD-grown wild-type, AtML1:LOG4 and AtML1:CKX1 plants. Scale bar = 50 μm. **(B)** Median longitudinal sections of the inflorescence meristems of LD-grown wild-type, AtML1:LOG4 and AtML1:CKX1 plants. Scale bar = 50 μm. **(C,D)** Diameter of the vegetative (**C**; *n* = 4–5) and inflorescence meristem (**D**; *n* = 4–6). **(E,F)** Leaf initiation rate under LD (**C**; *n* = 28–30) and SD conditions (**D**; *n* = 17–20). **(G)** Stem diameter (*n* = 17–24). Asterisks indicate significant differences compared to the wild type, as calculated by one-way ANOVA, *post hoc* Dunnett’s test **(C,D,G)** or Kruskal–Wallis test, *post hoc* Dunn’s test **(E,F)** (*p < 0.05; **p < 0.01; ***p < 0.001).

The CK status substantially influences the size and activity of the SAM as well as the size of the organs originating from it. A higher CK status leads to an increase in SAM size with more cells and delayed differentiation (Bartrina et al., 2011; Niemann et al., 2015), whereas CK deficiency results in the formation of smaller SAMs because of a reduced cell number and earlier differentiation (Werner et al., 2003; Higuchi et al., 2004; Nishimura et al., 2004; Tokunaga et al., 2012). To study if the CK originated from the L1 layer of the meristem controls its size, we compared the morphology of the vegetative and inflorescence meristems in the wild type and transgenic lines with the perturbed CK content. Figures 3A,C show that AtML1:LOG4 plants displayed an increase in the size of the vegetative meristem, whereas the SAMs of the AtML1:CKX1 lines were smaller compared to the wild type (Figures 3A,C). The extent of changes correlated with the expression level of the transgenes (Figures 1C,D). Similar although smaller changes were also detected in the inflorescence meristem (Figures 3B,D).

In order to investigate the activity of the vegetative SAM, we determined the plastochrone index, which is defined as the number of days a plant needs to produce one leaf. Given that CK integrates light signals during shoot organ formation (Yoshida et al., 2011), we carried out the analysis under different light regimes. Interestingly, we did not observe strong plastochrone differences under long day (LD) conditions (Figure 3E). However, correlating with the reduced meristem size, the leaf production rate was decreased in AtML1:CKX1 plants under short day (SD) conditions (Figure 3F).

SAM size is positively correlated with stem diameter (Schnablovà et al., 2017) and corresponding changes were noted in the transgenic lines (Figure 3G). The AtML1:LOG4 lines showed an increase in stem diameter of about 9% compared to the wild type and stem thickness was reduced to 68% and 89% of the wild type in the AtML1:CKX1 lines. The stem diameter of AtML1:ARR1-SRDX lines was comparable to the wild type (Figure 3G).

### Altered Epidermal CK Metabolism Alters Leaf Cell Number and Size

**FIGURE 4:**
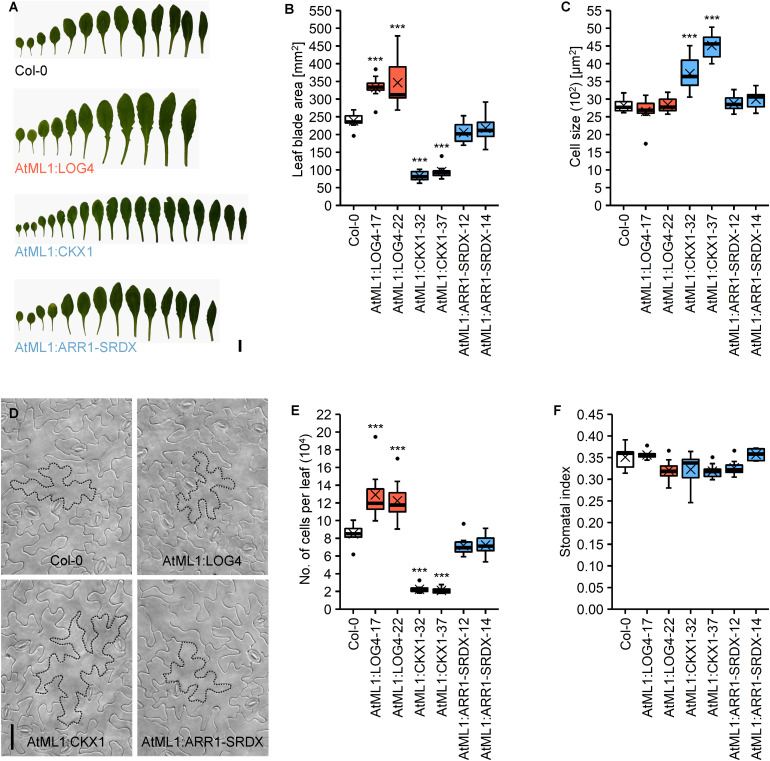
Analysis of the abaxial epidermis of the seventh rosette leaf of LD-grown *AtML1* lines. **(A)** Morphology of full grown leaves of Col-0, AtML1:LOG4-22, AtML1:CKX1-32, and AtML1:ARR1-SRDX-12 plants; scale bar = 1 cm. **(B)** Leaf blade size (*n* = 8). **(C)** Cell size (*n* = 8). **(D)** Abaxial epidermis of Col-0, AtML1:LOG4-22, AtML1:CKX1-32, and AtML1:ARR1-SRDX-12 leaves; exemplary cells are outlined with dotted lines; scale bar = 50 μm. **(E)** Number of cells per leaf (*n* = 8). **(F)** Stomatal index, defined as proportion of stomata within the total population of abaxial epidermal cells (*n* = 8). Asterisks indicate significant differences compared to the wild type, as calculated by one-way ANOVA, *post hoc* Dunnett’s test (*p < 0.05; **p < 0.01; ***p < 0.001).

The altered rosette size of transgenic plants (Figure 1A) led us to compare leaf development in more detail. In accordance with the changes in rosette size, the leaf blades of AtML1:LOG4 and AtML1:CKX1 plants were significantly bigger and smaller compared to the wild type, respectively (Figures 4A,B). The blade surface area of the seventh leaf in AtML1:LOG4 showed up to 45% increase, whereas AtML1:CKX1 leaves reached only 35–40% of the wild-type size. *AtML*1*:ARR1-SRDX* expression caused a significantly weaker, yet reproducible reduction of leaf blade size (Figure 4B). Since the differences in leaf size could be due to altered cell proliferation or expansion, we examined cell number and leaf cell size. Epidermal cell size in the AtML1:LOG4 leaves was similar to wild type, indicating that the enlarged leaf blade area resulted from increased cell proliferation and a larger total number of leaf cells. In contrast, the AtML1:CKX1 leaves contained only about a quarter of the cells of the wild type (Figure 4E), and the reduced cell number was partially compensated by an up to 60% enhancement of cell expansion (Figures 4C,D). These results are consistent with the role of CK in sustaining the cell proliferation phase by delaying cell differentiation (Holst et al., 2011; Skalák et al., 2019) and suggest that the epidermis-derived CK is relevant for this regulation. Reduced CK signaling output in the epidermis by the *AtML1*:*ARR1-SRDX* expression caused only a weak reduction of cell proliferation (Figures 4B–D), suggesting that the leaf growth control involves largely a non-cell-autonomous CK activity in the epidermis.

Finally, we took a closer look on the distribution of stomata, which are a specialized cell type in the lower epidermis. The stomatal index, which is defined as the proportion of stomata in the total population of epidermal cells, can provide information about possible influences of the altered CK status on the differentiation of these cells. However, neither the altered CK metabolism in the AtML1:LOG4 and AtML1:CKX1 nor the reduced CK signaling in the AtML1:ARR1-SRDX lines changed the stomatal index (Figure 4F) suggesting that this parameter is not under control of CK.

### Altered Epidermal CK Metabolism Impacts Developmental Transitions

**FIGURE 5:**
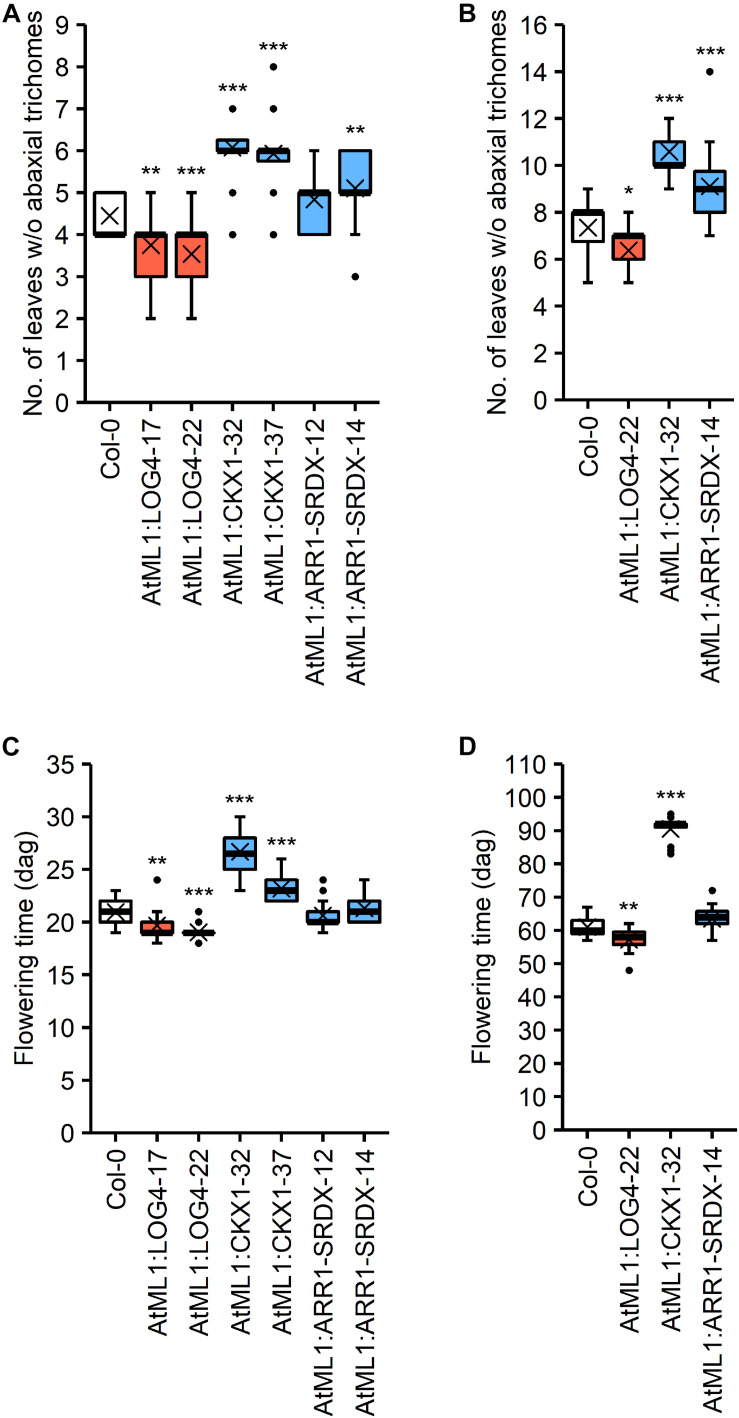
Impact of the expression of *AtML1*:*LOG4, AtML1*:*CKX1*, and *AtML1:ARR1-SRDX* on developmental transitions. **(A,B)** Number of leaves without abaxial trichomes under LD (**A**; *n* = 28–30) and SD conditions (**B**; *n* = 17–20). **(C,D)** Flowering time of *AtML1* transgenic lines compared to the wild type under LD (**A**; *n* = 28–30) and SD conditions (**B**; *n* = 19–20). Asterisks indicate significant differences compared to the wild type, as calculated by one-way ANOVA, *post hoc* Dunnett’s test (*p < 0.05; **p < 0.01; ***p < 0.001).

CK has an influence on the juvenile-to-adult phase transition (Kiba et al., 2013; Sören Werner, Isabel Bartrina, Thomas Schmülling, unpublished result) as well as on flowering time (Michniewicz and Kamieńska, 1965; Werner et al., 2003; Nishimura et al., 2004; Riefler et al., 2006). The epidermis-specific modulation of CK metabolism and signaling impacted these developmental transitions (Figure 5). The transgenic lines were grown under SD and LD conditions and the number of leaves without abaxial trichomes as a measure for juvenile leaf number (Telfer et al., 1997) as well as the time until bolting were examined.

The higher CK status in AtML1:LOG4 lines resulted in an earlier transition from the juvenile to the adult phase under both LD and SD, whereas the reduction of CK content in the AtML1:CKX1 lines led to significant increases in juvenile leaf number (Figures 5A,B). Interestingly, the juvenile-to-adult transition was significantly affected by the *AtML1*:*ARR1-SRDX* transgene (Figure 5B), suggesting an at least partly epidermis-autonomous control of this process by CK.

Transition to flowering occurred earlier in AtML1:LOG4 plants than in wild type and was delayed in AtML1:CKX1 under both LD and SD conditions (Figures 5C,D). In contrast, the flowering time of AtML1:ARR1-SRDX plants was unchanged in comparison to wild type (Figures 5C,D), suggesting that the mobile nature of the signal controlling the developmental transition to flowering is relevant.

### Expression of *AtML1*:*LOG4* and *AtML1*:*CKX1* Influences Seed Yield

**FIGURE 6:**
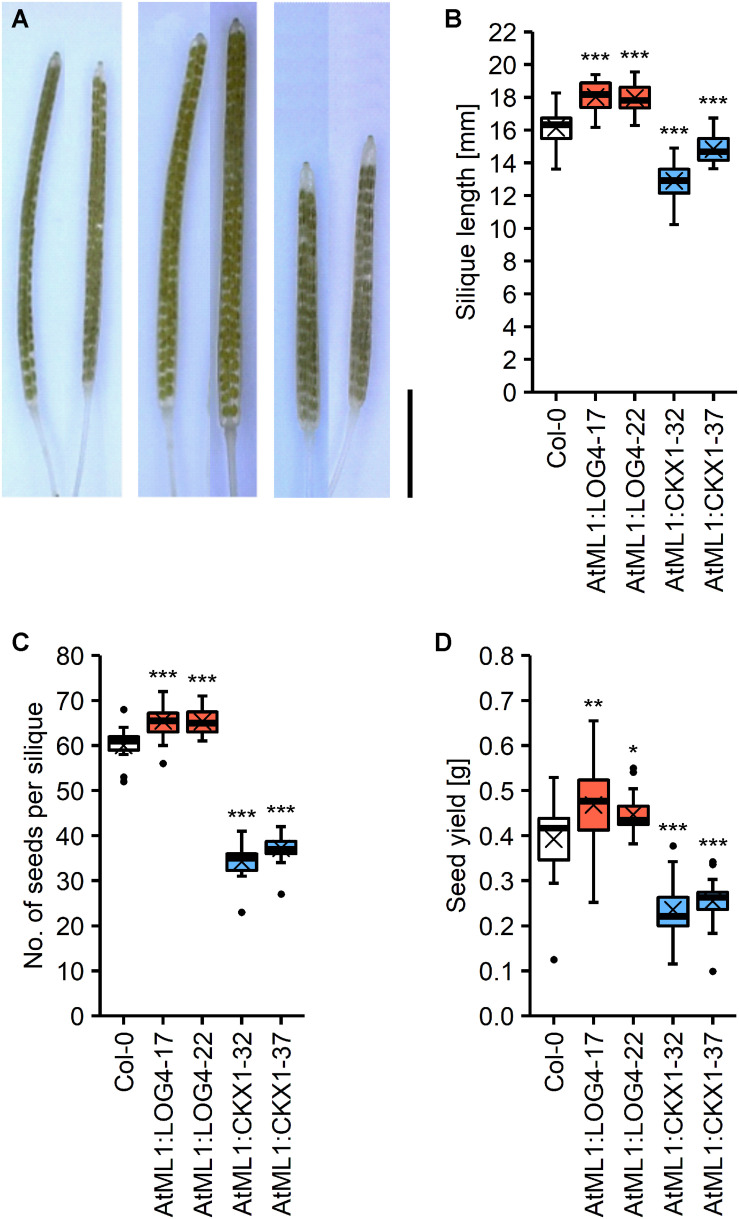
Expression of *AtML1*:*LOG4* and *AtML1*:*CKX1* influences seed yield. **(A)** Cleared siliques; order from left to right: Col-0, AtML1:LOG4-22, AtML1:CKX1-32 (scale bar = 5 mm). **(B)** Silique length (*n* = 18–30). **(C)** Number of seeds per silique (*n* = 18–30). **(D)** Seed yield per plant (*n* = 24–25). Asterisks indicate significant differences compared to the wild type, as calculated by one-way ANOVA, *post hoc* Dunnett’s test (*p < 0.05; **p < 0.01; ***p < 0.001).

CK has a strong impact on reproductive development of *Arabidopsis* (Bartrina et al., 2011; Werner et al., 2003; Zúñiga-Mayo et al., 2018, 2019). In order to explore the potential relevance of CK of the epidermal layer in regulating reproductive traits we compared various parameters of reproductive growth and development between the transgenic lines and wild type. The flowers and gynoecia of lines with a reduced and increased CK status were smaller and bigger, respectively, than wild type. However, these differences were not in all cases statistically significant at the developmental stage that was evaluated (Supplementary Figure S2). The siliques of AtML1:LOG4 and AtML1:CKX1 lines were bigger and smaller, respectively, showing that transgene expression influences growth processes of the reproductive organs (Figures 6A,B). The altered silique sizes in these lines correlated with a higher and lower number of seeds developed per silique, respectively (Figure 6C), indicating an impact of transgene expression on ovule formation during gynoecia development. The seed yield per plant was increased in the AtML1:LOG4 lines by 9% and decreased in AtML1:CKX1 lines by 41% (Figure 6D).

In order to investigate if the impact of transgene expression on seed yield can be attributed to the altered silique length alone, we analyzed other growth parameters contributing to seed yield. AtML1:CKX1 plants produced about 16% bigger seeds as compared to wild type (Supplementary Figures S3A,B), nonetheless showing a lower total seed weight than wild type (Figure 6D). Among the transgenic lines, only AtML1:CKX1 plants exhibited a reduced number of siliques at the main stem (Supplementary Figure S3C), but the average silique number per plant was not altered due to an apparently reduced apical dominance and strongly increased number of axillary branches (Supplementary Figures S3D,E). Plant height was affected in AtML1:LOG4 plants, which grew 16% larger than wild type (Supplementary Figure S3F), however, the total number of siliques remained similar to wild type (Supplementary Figures S3C,D). In AtML1:ARR1-SRDX plants all these parameters were similar to wild type (Supplementary Figures S3C–F) indicating that abbreviation of CK signaling output in the epidermal cell layer has no impact on silique number, branching, and plant height. It can be concluded that the impact of *AtML1*:*LOG4* and *AtML1*:*CKX1* expression on seed yield is mainly attributable to the altered number of seeds harbored by individual pods.

### AHK2, AHK3, and ARR1 Mediate the Effect of Epidermal CK

**FIGURE 7:**
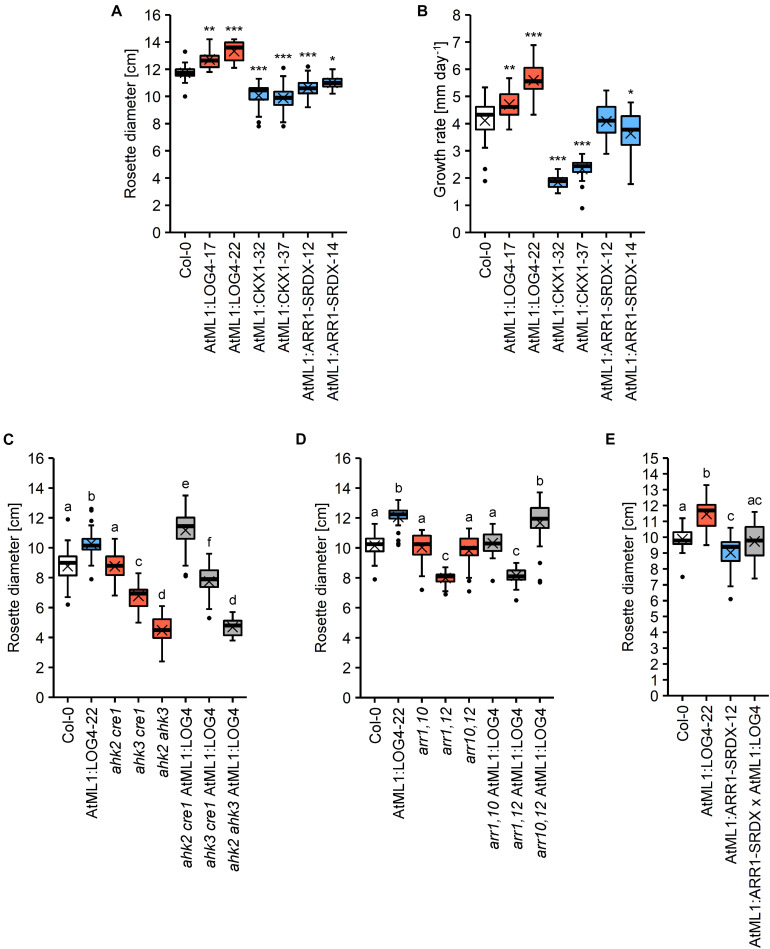
Rosette growth of *AtML1* lines. **(A)** Rosette diameter (*n* = 18–24) and **(B)** growth rate of the rosette (*n* = 26–27) of *AtML1* lines. Asterisks indicate significant differences compared to the wild type, as calculated by one-way ANOVA, *post hoc* Dunnett’s test (*p < 0.05; **p < 0.01; ***p < 0.001). **(C)** Influence of CK receptor mutations on the enhanced rosette growth of AtML1:LOG4 plants grown under LD conditions for 33 days (*n* = 28). **(D)** Influence of mutations of type-B response regulators on the enhanced rosette growth of AtML1:LOG4 plants grown under LD conditions for 29 days (*n* = 27–28). Letters indicate significant differences between the genotypes, as calculated by one-way ANOVA, *post hoc* Tukey’s test (*p* < 0.05). **(E)** Rosette diameter of LD-grown AtML1:ARR1-SRDX AtML1:LOG4 plants (28 DAG) compared to the parental lines (*n* = 21–24).

Next we were interested to investigate through which signaling pathway the epidermis-derived CK signal is transmitted. To this end, the stronger expressing AtML1:LOG4-22 line (Figure 1C) was introgressed into three CK receptor double mutants (*ahk2 cre1*, *ahk3 cre1*, and *ahk2 ahk3*) as well as into double mutants of the type-B response regulator genes *ARR1*, *ARR10*, and *ARR12* (*arr1,10*, *arr1,12*, and *arr10,12*). Supplementary Figure S4 shows that the transgene was expressed to a similar level in the different mutant backgrounds. As a parameter to evaluate the genetic interaction, we chose terminal rosette diameter, which was significantly altered in all transgenic lines. On average, AtML1:LOG4 lines had an 11% increase in rosette size (13.6% in the stronger expressing line AtML1:LOG4-22), AtML1:CKX1 showed a reduction of about 15% and the AtML1:ARR1-SRDX lines exhibited a decrease in rosette diameter of 8% in comparison to the wild type (Figure 7A). Corresponding changes in rosette growth rate were detected in the respective transgenic lines (Figure 7B).

In the wild-type background, the *ahk2 cre1* double mutant had no impact on rosette diameter, *ahk3 cre1* showed a 22.6% reduction and *ahk2 ahk3* showed with 48.8% reduction the strongest change compared to wild type. Expression of *AtML1:LOG4* caused an increase of the rosette diameter in *ahk2 cre1* indicating that AHK3 alone is sufficient to transmit the CK signal (Figure 7C). AtML1:LOG4 *ahk3 cre1* hybrids showed a reduction of rosette diameter as compared to AtML1:LOG4 which was even stronger in the AtML1:LOG4 *ahk2 ahk3* hybrids. AtML1:LOG4 significantly enhanced the rosette diameter of *ahk3 cre1*, whereas no effect was observed in *ahk2 ahk3* mutants (Figure 7C). This suggests also a partial contribution of AHK2 to mediate the CK effect while CRE1/AHK4 had apparently no role.

*ARR1*, *ARR10*, and *ARR12* are among the most highly expressed type-B *ARR* genes in leaves (Mason et al., 2004; Tajima et al., 2004) and they mediate most if not all of the effects of CK on vegetative growth (Argyros et al., 2008; Ishida et al., 2008). To test their involvement in the enhanced rosette growth observed in AtML1:LOG4, we introgressed the *AtML1:LOG4* transgene into all three double mutant combinations. The transgene expression enhanced the rosette size only in the background of *arr10,12* (Figure 7D), suggesting an involvement of ARR1 in mediating rosette growth by epidermal CK.

### CK Signaling From the Epidermis Promotes Leaf Growth

Increased CK biosynthesis by *AtML1*:*LOG4* expression enhanced leaf growth. This could be due to a cell-autonomous effect of CK in the epidermis, and/or due to a growth stimulatory effect of epidermally produced CK on the underlying tissues. To address this question experimentally, we suppressed the CK signaling in the epidermis of AtML1:LOG4 plants by crossing to AtML1:ARR1-SRDX. The rosette diameter of a homozygous hybrid line expressing both transgenes (Supplementary Figure S5) was analyzed. Figure 7E shows that the rosette size of the transgenic hybrid was comparable to wild type, indicating that the growth promoting effect of *AtML1*:*LOG4* was abolished. Hence, the results suggest that CK signaling in the epidermal layer promotes leaf growth in a, at least partially, cell-autonomous fashion.

## Discussion

Enhanced CK biosynthesis or breakdown in the epidermis caused numerous developmental changes of shoot growth that are known from *Arabidopsis* mutants and transgenic plants with an altered CK content or signaling (Werner et al., 2003; Higuchi et al., 2004; Nishimura et al., 2004; Riefler et al., 2006; Argyros et al., 2008; Ishida et al., 2008; Holst et al., 2011; Bartrina et al., 2011, 2017; Niemann et al., 2015; Skalák et al., 2019). These changes included an altered size of the shoot apical meristem, altered leaf and rosette size, altered flowering time, and an impact on seed size and seed yield. This indicates that CK breakdown and CK synthesis in the epidermis can be a factor regulating various aspects of shoot development. The fact that numerous CK metabolism and signaling genes are expressed in the epidermis (Yadav et al., 2014) is consistent with this conclusion.

It needs to be considered that CK is transported and some CK metabolites might also diffuse to surrounding cells or reach them through plasmodesmata. Therefore, it is likely that enhanced degradation of CK in the epidermis weakened its function as source of the hormone and thus indirectly affected tissues adjacent to the epidermis. In an opposite scenario, it cannot be excluded that enhanced CK degradation in the epidermis drained CK from underneath tissues. Similarly, enhanced CK synthesis in the epidermis could act in the epidermis itself or in deeper cell layers. In fact, CK synthesized in the L1 has been proposed to form gradients over several cell layers to regulate *WUS* expression in the organizing center (Chickarmane et al., 2012; Gruel et al., 2016). Further, other work has shown that CK regulating the SAM might come from the young leaf primordia (Holst et al., 2011) or even from roots (Osugi et al., 2017) documenting the mobile nature of the hormone. Notably, a non-cell-autonomous effect of CK was also observed when CK metabolism was experimentally altered during gynoecium development (Marsch-Martínez et al., 2012). Taken together, the phenotypic changes caused by expression of *AtML1*:*CKX1* and *AtML1*:*LOG4* must not be or at least not entirely be due to events in the epidermis itself. Consistently, the consequences of *AtML1*:*ARR1-SRDX* expression were generally smaller or even absent as compared to those of *AtML1:CKX1*.

If and to which extent CK acts in the epidermis itself is probably best illustrated by the analysis of leaf and rosette size (reflecting leaf length) of the transgenic lines. CK has numerous functions during leaf development. It regulates cell division during the early phase, the transition of cells from the division to the differentiation phase, the following cell enlargement, and finally termination of leaf development (Werner et al., 2003; Holst et al., 2011; Efroni et al., 2013; Bartrina et al., 2017; Skalák et al., 2019). It has been shown that it is sufficient to alter the CK content during the early cell division phase of leaf development to obtain profound effects on leaf size indicating that its effect on cell division and the inhibition of cell differentiation are most relevant for regulating leaf size (Holst et al., 2011; Skalák et al., 2019).

Both AtML1:CKX1 and AtML1:ARR1-SRDX plants had a reduced leaf and rosette size compared to wild type. The leaf surface in AtML1:CKX1 plants was reduced by about 65% and the rosette diameter by about 15%, while the size reduction was smaller in AtML1:ARR1-SRDX plants reaching on the average 89% of leaf size and 92% of the rosette diameter of wild type. Notably, the size reduction of AtML1:CKX1 leaves was less strong than in leaves of 35S:CKX1 and 35:CKX3 plants (Werner et al., 2003; Holst et al., 2011). Similarly, systemically reduced CK signaling in 35S:ARR1-SRDX plants had a much stronger effect on leaf size (Heyl et al., 2008) than epidermal expression of the transcriptional repressor in AtML1:ARR1-SRDX. The stronger consequences of systemic interference with CK metabolism or signaling suggests that the CK status of deeper cell layer is also a relevant factor in regulating leaf growth.

Furthermore, cells of the abaxial epidermis of AtML1:CKX1 plants were 30–60% larger than in wild type indicating that a compensatory effect had been initiated. An increase in cell size to compensate for a lower cell number is known from CK-deficient plants (Werner et al., 2003; Holst et al., 2011; Skalák et al., 2019) as well as from plants with altered cell cycling (reviewed by Horiguchi and Tsukaya, 2011). In contrast, the size of cells in the epidermis of AtML1:ARR1-SRDX and AtML1:LOG4 leaves was similar to wild type, but leaves of AtML1:LOG4 plants formed about 35% more cells. We conclude that epidermal CK metabolism impacts cell division and the transition to cell differentiation thus regulating leaf cell number and leaf size. Notably, the increase in cell size in AtML1:CKX1 leaves was similar to 35S:CKX3 leaves and larger than in ANT:CKX3 leaves (Holst et al., 2011). This supports the conclusion that CK restricts cell enlargement during leaf expansion growth when the *AtML1* and *35S* promoters are still active but the primordia-specific *ANT* promoter is no longer expressed.

The relevance of epidermal CK acting in the epidermis itself to regulate leaf size might be concluded from the phenotype of AtML1:LOG4/AtML1:ARR1-SRDX hybrid plants. In these plants, the promotive influence of CK synthesis in the epidermis on leaf size was strongly suppressed by abbreviating CK signaling in this cell layer. Principally, the LOG4-driven CK production in the epidermis could still increase CK concentrations in deeper tissues but this had apparently no strong influence on leaf size. Thus, the hybrid phenotype indicated that CK synthesized in the epidermis acted on epidermal cells delaying their differentiation. This example clearly illustrates that CK promotes leaf growth in the epidermal layer in an at least partially layer-autonomous fashion.

Genetic analysis showed that the AHK2 and AHK3 receptors together with the transcription factor ARR1 mediate the CK effect on rosette growth in AtML1:LOG4 plants as mutation of these genes abolished the LOG4-induced growth enhancement. *LOG4* expression in the *ahk2 ahk3* background had no effect on leaf size at all consistent with the known relevance of these receptors in regulating leaf traits (Higuchi et al., 2004; Nishimura et al., 2004; Riefler et al., 2006; Cortleven et al., 2014), their strong signaling activity in leaves (Stolz et al., 2011), and their expression in the epidermis (Yadav et al., 2014). Furthermore, AHK2 and AHK3 are prime candidates to perceive the CK signal in the cell internal membrane system (Romanov et al., 2006; Wulfetange et al., 2011; Antoniadi et al., 2020), which may be required for the cell-autonomous activity of CK in the epidermis. Mutation of *ARR1* also blocked completely the response to *AtML1:LOG4* and ARR1 alone was sufficient to mediate the growth enhancement by CK production in the epidermis in the *arr10,12* mutant. This indicates a singular role for this transcription factor in mediating CK activity in the epidermis. The more drastic growth reduction of the rosettes in *ahk2 ahk3 cre1* and *arr1,10,12* triple mutants (Nishimura et al., 2004; Higuchi et al., 2004; Riefler et al., 2006; Argyros et al., 2008; Ishida et al., 2008) suggests that CRE1/AHK4, ARR10, and ARR12 also have functions in regulating leaf growth but probably not in the epidermal cell layer.

CK synthesized in the epidermis of AtML1:LOG4 plants promoted the juvenile-to-adult phase transition while increased CK breakdown or reduced signaling caused a later phase transition (Figures 5A,B). Vegetative phase change relies strongly on the balance of miR156 and miR172 and the activity of their respective target genes (Huijser and Schmid, 2011; Poethig, 2013). The abaxial trichomes formed on adult leaves are a commonly used indicator for the transition from the juvenile to the adult phase (Telfer et al., 1997) and they are derived from the epidermis. Thus it could be that the hormone exerts its activity in part directly in the layer where it is produced, which is consistent with the later phase transition of AtML1:ARR1-SRDX. Their weak phenotype compared to AtML1:CKX1 suggests that not the epidermis alone, but rather the whole leaf determines epidermal identity. The regulation of trichome formation from mesophyll depending on the transcription factor TEMPRANILLO shows that there are (also) actions from a distance (Matías-Hernández et al., 2016). Whether CK also affects other leaf traits accompanying vegetative phase change needs to be determined.

Another developmental transition, the onset of flowering showed a different picture. Increased CK synthesis and CK breakdown in the epidermal cell layer caused earlier and later flowering, respectively (Figures 5C,D). This is consistent with the known promotive effect of CK on flowering in *Arabidopsis* (Kieber and Schaller, 2018). Abbreviating CK signaling by epidermal expression of *AtML1*:*ARR1-SRDX* did not alter flowering time suggesting that the CK status of the epidermis itself has no influence on flowering but that altered epidermal CK metabolism influenced the activity of a mobile signal promoting the transition to flowering. Given the long-distance transport of CK in plants, it can be hypothesized that this mobile signal is CK itself.

The higher seed yield of AtML1:LOG4 plants shows that a limited and layer-specific increase in CK content may cause a higher seed yield. This increase was mainly due to the higher number of seeds harbored by individual siliques which were larger than in wild type. Consistently, AtML1:CKX1 plants had smaller siliques with less seeds. Furthermore, the difference in seed yield may be related to the number of ovules that were initiated in the gynoecia. Indeed, CK promotes the activity of the placenta and regulates the ovule number and the distance between ovules (Bartrina et al., 2011; Bencivenga et al., 2012; Cerbantez-Bueno et al., 2020) and *AtML1* is expressed in the placenta (Lu et al., 1996). Interestingly, other yield traits previously shown to be regulated by CK such as the activity of the inflorescence meristem and the duration of flowering (Bartrina et al., 2011, 2017) were not regulated by epidermal CK. In any case, the demonstration that expression of *AtML1:LOG4* as a single dominant gene increases seed yield is of potential biotechnological interest as yield traits are also controlled by CK in oilseed rape (*Brassica napus* L.), which is an important source for vegetable oil and close relative of *Arabidopsis* (Zúñiga-Mayo et al., 2018; Schwarz et al., 2020).

Collectively, the analysis has shown that manipulating the CK metabolism in the epidermis can alter plant growth and development. It is clear from the analysis of leaf size regulation that layer-autonomous activity of CK affects primarily cell division and differentiation. This contrasts with brassinosteroid and ethylene, which both regulate cell expansion of epidermal cells (Savaldi-Goldstein et al., 2007; Vaseva et al., 2018) in a layer-autonomous fashion. Clearly, further experiments are required to resolve the spatial organization of CK action and its potential role in coordinating cell division and differentiation of the epidermal cells and cells of underlying tissues.

## Materials and Methods

### Plant Material and Growth Conditions

The Columbia-0 (Col-0) ecotype of *Arabidopsis thaliana* served as the wild type. All mutants used in this study are listed in Supplementary Table S2. All genotypes were confirmed by PCR analysis and the primers used for genotyping are listed in Supplementary Table S3. For all experiments conducted on soil, sown *Arabidopsis* seeds were kept at 4°C for 2 days in the dark and then exposed to LD (16 h light/8 h dark cycle) or SD conditions (8 h light/16 h dark cycle), at 22°C and 60% humidity and a light intensity of 100–150 μmol m^–2^ s^–1^. Individuals of different lines were randomized to minimize positional effects. For *in vitro* experiments, seeds were surface sterilized using a 1.2% (v/v) sodium hypochlorite/0.01% (v/v) Triton X-100 solution. After stratification, seedlings were grown at 22°C on 1/2 × Murashige and Skoog (MS) medium containing 0.22% (w/v) MS basal salt, 0.1% (w/v) sucrose, 0.05% (w/v) MES, and 0.8% (w/v) agar (pH adjusted to 5.7).

### Plasmid Constructions

For the generation of *AtML1*:*GFP-GUS*, *AtML1*:*LOG4*, *AtML1*:*CKX1*, and *AtML1*:*ARR1-SRDX* constructs, the Gateway^®^ system (ThermoFisher, Waltham, MA, United States) was used. All primers used for cloning purposes are listed in Supplementary Table S4. A 3.9 kb long *AtML1* promoter fragment (−5488 to −1581 bp upstream of the *AtML1* start codon) was amplified from Col-0 genomic DNA using Phusion High-Fidelity DNA-Polymerase (ThermoFisher). The fragment was linked to the Gateway^®^ attB1/attB2 and attB4/attB1R sites by PCR and cloned into pDONR221 and pDONR P4-P1R, respectively, using Gateway BP Clonase Enzyme Mix (ThermoFisher). The pDONR221/*pAtML1* vector was used to generate a transcriptional fusion with the *GUS* gene in pKGWFS7 (Karimi et al., 2002) using Gateway LR Clonase Enzyme Mix (ThermoFisher). The *LOG4* gene coding sequence was amplified from Col-0 cDNA including the stop codon. For *CKX1*, the genomic sequence from Col-0 lacking the stop codon was used for cloning. The protein coding region of *A. thaliana* accession C24 *ARR1* fused to the SRDX peptide (LDLDLELRLGFA) was amplified from pDONR201/ARR1-SRDX (Heyl et al., 2008) without stop codon. All gene fragments were cloned first into pDONR221 and then combined with *AtML1*-attB4/attB1R in pB7m34GW (Karimi et al., 2005) using LR Clonase II Enzyme Mix (ThermoFisher). The third position at the 3′ end in pB7m34GW was filled with a 4xMyc tag, creating a translational fusion in case of *CKX1* and *ARR1-SRDX*. All cloned DNA fragments were fully sequenced to exclude PCR errors. All plasmids were individually transformed into the *Agrobacterium tumefaciens* strain GV3101:pMP90 by electroporation and the resulting strains were subsequently used to transform *Arabidopsis* plants using the floral-dipping method (Clough and Bent, 1998).

### RNA Preparation and Quantitative RT-PCR

Approximately 100 mg of plant material was harvested and frozen in liquid nitrogen at the indicated time points. The frozen samples were ground using a Retsch mill in precooled adapters. Total RNA was extracted using TRIsure^TM^ (Bioline) following the manufacturer’s instructions. The RNA pellet was resuspended in 40–50 μl of nuclease-free water and treated with DNase I (ThermoFisher), following the manufacturer’s instructions. For cDNA synthesis, 1.0–1.5 μg of total RNA was reverse transcribed using SuperScript^TM^ III (ThermoFisher), 4.5 μM of N9 random oligos, and 2.5 μM of oligo-dT_25_ in a 20 μl reaction. Mix 1 containing RNA, 2 mM of dNTP mix and oligos was incubated for 5 min at 65°C and placed on ice afterwards. Mix 2 (first strand buffer, 5 mM DTT, SuperScript^TM^ III) was added and samples were incubated for 30 min at 25°C, 60 min at 50°C, and 15 min at 70°C. The resulting cDNA was diluted 1:5. For qRT-PCR analysis, *PROTEIN PHOSPHATASE 2A SUBUNIT A2 (PP2AA2)* and *TBP-ASSOCIATED FACTOR II 15 (TAFII15)* served as reference genes. All qRT-PCR primers used in this study are listed in Supplementary Table S5. qRT-PCR was performed with the 7500 Fast RealTime PCR system (Applied Biosystems) using SYBR Green I technology and universal FAST cycling conditions (15 min at 95°C, 40 cycles of 5 s at 95°C, 15 s at 55°C, and 10 s at 72°C) followed by the generation of a dissociation curve to check for specificity of the amplification. Gene expression data analysis was carried out according to Vandesompele et al. (2002). For analysis of *ARR1-SRDX* transgene expression, the 40−Δ*Ct* method was used, as described by Morcuende et al. (2007).

### Cytokinin Measurements

For the determination of CK metabolites, plants were grown on soil under LD conditions. Approximately 20 mg of shoot material of 7-day-old seedlings were pooled, frozen in liquid nitrogen and stored at −80°C until further analysis. Four independent samples were analyzed for each genotype. The CK content was determined by ultraperformance liquid chromatography-electrospray tandem mass spectrometry (Novák et al., 2008).

### Phenotypic Analyses

Plant height and rosette diameter were measured with a ruler. Plant rosette size was determined by measuring the maximal rosette diameter after progression to the reproductive phase. For the analysis of the rosette growth rate, the rosette diameter was measured every 2–3 days. The calculation of the growth rate was based on the linear growth phase. Flowering time was defined as the day the inflorescence stem was 0.5 cm long. Silique, flower and gynoecium length as well as SAM and seed diameter were measured using ImageJ (Abràmoff et al., 2004). To determine the size of the vegetative and inflorescence meristems, the distance at the base of the SAM between the primordia was measured. For the determination of seed size, length and width of every seed were measured and the mean of these values is shown as the average seed diameter.

### Histology and Histochemistry

For the determination of cell size, cell number, and stomatal index, the seventh rosette leaves were cleared (Malamy and Benfey, 1997) and analyzed by a stereomicroscope (SZX12; Olympus, Tokyo, Japan) and a microscope (Axioskop 2 plus with AxioCam ICc3 camera; Zeiss, Jena, Germany). The whole leaf blade area was measured using the ImageJ plugin LeafJ (Maloof et al., 2013) and the number of pavement and guard cells per defined area at six different positions of the abaxial epidermis were determined from digital micrographs: at 25%, 50%, and 75% distance between the base and the tip of the leaf blade, in the midst of the leaf margin and the main vein on both sides. From these data, the number of cells per leaf, average cell size, and stomatal index were calculated. To conduct tissue sections, plant material was vacuum-infiltrated with fixative solution containing 10% (v/v) formaldehyde, 5% (v/v) acetic acid, and 50% (v/v) ethanol. After incubation at 4°C for 8–12 h, plant material was dehydrated by subjecting it to 50%, 60%, 70%, 85%, 95%, and 100% ethanol for 45 min each at 4°C. Samples were incubated in ethanol with increasing amounts of Roti^®^-Histol (Carl Roth, Karlsruhe, Germany) at room temperature. Paraplast X-TRA^®^ (Sigma-Aldrich, Munich, Germany) was added to the vials and incubated at 58°C overnight. Wax was changed several times for 3 days and ultimately was solidified at room temperature. Cross sections were performed at a rotary microtome (RM2255, Leica, Wetzlar, Germany). For the analysis of the stem diameter, stem fragments were taken 2–3 cm above the rosette when the main inflorescence was 12–15 cm high. The stem segments were photographed using the SZX12 stereomicroscope and 14 μm thick cross sections were prepared. After staining with 0.1% toluidine blue, sections were analyzed with the Axioskop microscope. Stem diameter was measured using ImageJ (Abràmoff et al., 2004). GUS staining of *Arabidopsis* seedlings was performed according to Hemerly et al. (1993). The tissue was cleared for the microscopical analysis according to Malamy and Benfey (1997). For the confirmation of the epidermal expression, 8 μm tissue sections of the SAM area were produced and analyzed with the Axioskop microscope. For the analysis of SAM size, 30-day-old SD-grown plants as well as the inflorescences of LD-grown plants were fixed as described and 8 μm tissue sections were generated.

### Statistical Analysis

All data are expressed as mean ± SEM. Statistical analyses were performed using GraphPad Prism, version 8 (GraphPad Software, La Jolla, CA, United States). Statistical tests used are indicated in the figure and table legends. A *p*-value < 0.05 was considered to indicate a significant difference.

### Accession Numbers

Sequences of genes and intergenic regions described in this article can be found in The Arabidopsis Information Resource (http://www.arabidopsis.org/) under the following accession numbers: *AHK2* (AT5G35750), *AHK3* (AT1G27320), *CRE1/AHK4* (AT2G01830), *ARR1* (AT3G16857), *ARR4* (AT1G10470), *ARR5* (AT3G48100), *ARR6* (AT5G62920), *ARR7* (AT1G19050), *ARR9* (AT3G57040), *ARR10* (AT4G31920), *ARR12* (AT2G25180), *AtML1* (AT4G21750), *CKX1* (AT2G41510), and *LOG4* (AT3G53450).

## Data Availability Statement

The original contributions presented in the study are included in the article/Supplementary Material, further inquiries can be directed to the corresponding author/s.

## Author Contributions

SW, IB, TW, and TS developed the project. SW performed experiments. SW, IB, TW, and TS analyzed the data. ON and MS measured cytokinin concentrations. SW and TS wrote the manuscript. All authors contributed to the article and approved the submitted version.

## Conflict of Interest

The authors declare that the research was conducted in the absence of any commercial or financial relationships that could be construed as a potential conflict of interest. The reviewer SF declared a past co-authorship with one of the authors ON to the handling editor.
