# Supplementary material for: The Cytokinin Status of the Epidermis Regulates Aspects of Vegetative and Reproductive Development in Arabidopsis thaliana
Source: Front Plant Sci. 2021 Feb 23;12:613488. doi: 10.3389/fpls.2021.613488 (PMC7959818; doi:10.3389/fpls.2021.613488)
Supplement: Supplementary file 1 [file Data_Sheet_1.PDF]

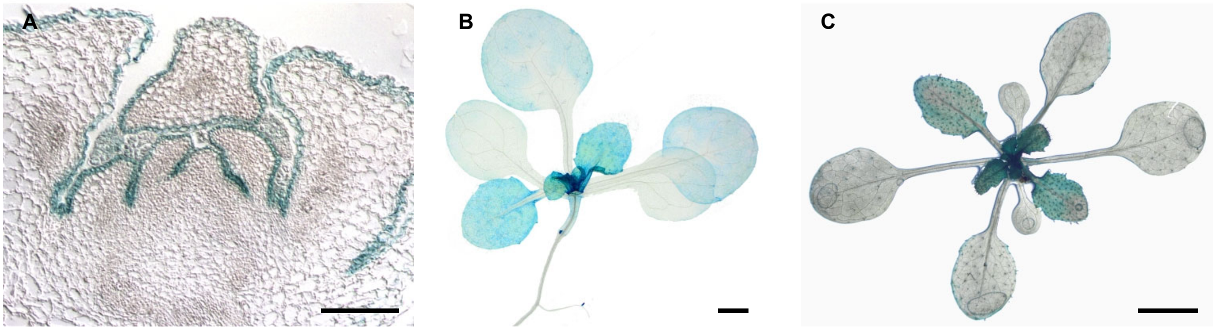

FIGURE S1 │ *AtML1* promoter activity in the shoot. (A) Longitudinal sections through the SAM region of 10-d-old GUS stained AtML1:GFP-GUS seedlings grown in LD (scale bar = 100 μm). **(B)** 12-d-old GUS stained AtML1:GFP-GUS seedlings grown in LD (scale bar = 1 mm). **(C)** 16-d-old GUS stained AtML1:GFP-GUS plants grown in LD (scale bar = 5 mm).

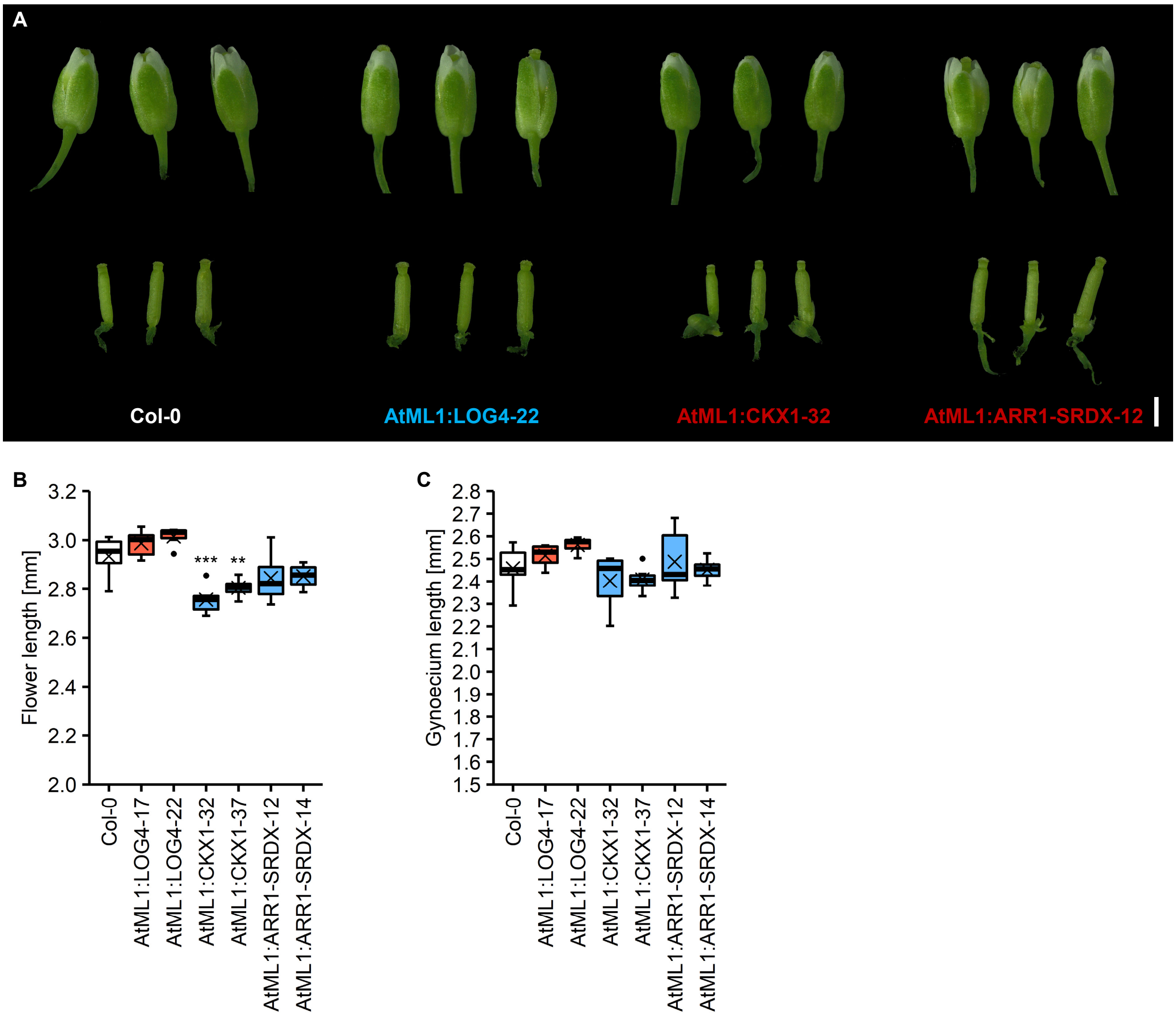

FIGURE S2 │ Flowers and gynoecia of *AtML1* lines. (A) Flowers (stage 13 after Smyth *et al.,* 1990) and gynoecia of LD-grown AtML1:LOG4, AtML1:ARR1-SRDX and AtML1:CKX1 compared to the wild type. Scale bar = 1 mm. **(B)** Average flower length (n = 6). **(C)** Average gynoecium length (n = 6). Asterisks indicate significant differences compared to the wild type, as calculated by One-Way ANOVA, post-hoc Dunnett‘s test (*, $p < 0.05$; **, $p < 0.01$; ***, $p < 0.001$).

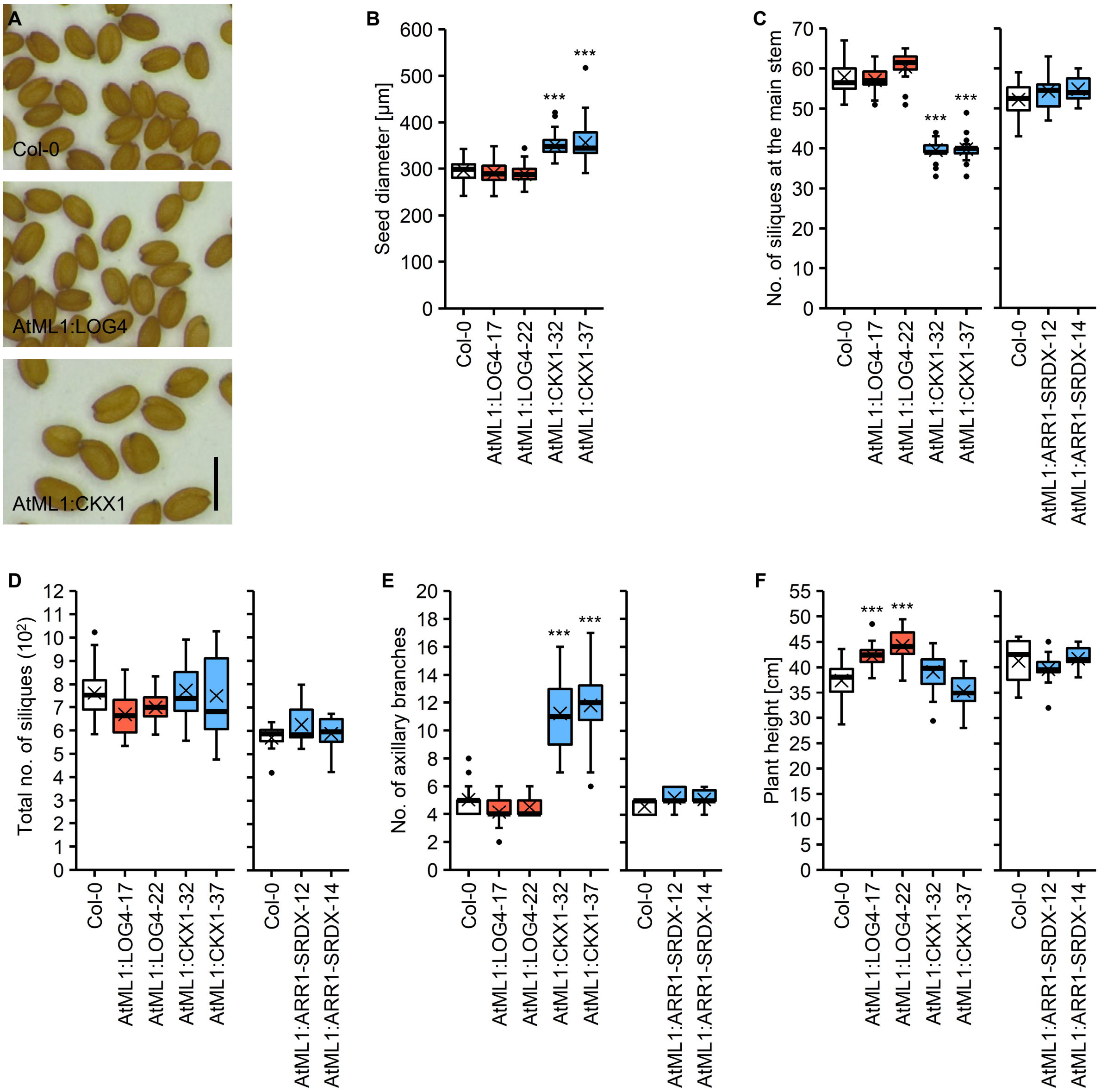

FIGURE S3 │ Number of siliques, plant height, number of axillary branches and seed size of LD-grown *AtML1* lines. (A) Seed morphology (scale bar = 500 µm). **(B)** Average seed diameter (n = 60). **(C)** Number of siliques at the main stem (left, n = 18-20; right, n = 10). **(D)** Total number of siliques (left, n = 18-20; right, n = 10). **(E)** Number of axillary branches (left, n = 18-20; right, n = 10). **(F)** Terminal plant height (left, n = 18-20; right, n = 10). Asterisks indicate significant differences compared to the wild type, as calculated by Kruskal-Wallis test, post-hoc Dunn‘s test (B) or One-Way ANOVA, post-hoc Dunnett‘s test (C-F) (*, $p < 0.05$; **, $p < 0.01$; ***, $p < 0.001$).

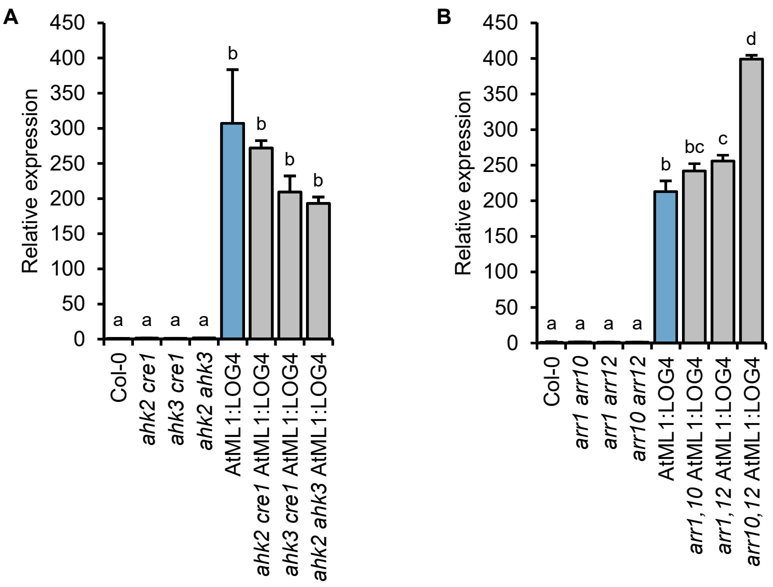

FIGURE S4 │ *AtML1:LOG4* transgene expression in the background of CK receptor and B-type *ARR* double mutants. Expression level of *LOG4* in shoots of 10-d-old LD-grown seedlings. Letters indicate significant differences between the genotypes, as calculated by One-way ANOVA, post-hoc Tukey's test ($p < 0.05$). Transcript levels were determined by qRT-PCR, data were normalized to *PP2AA2* and *TAFII15,* values are mean ± SEM, n = 4.

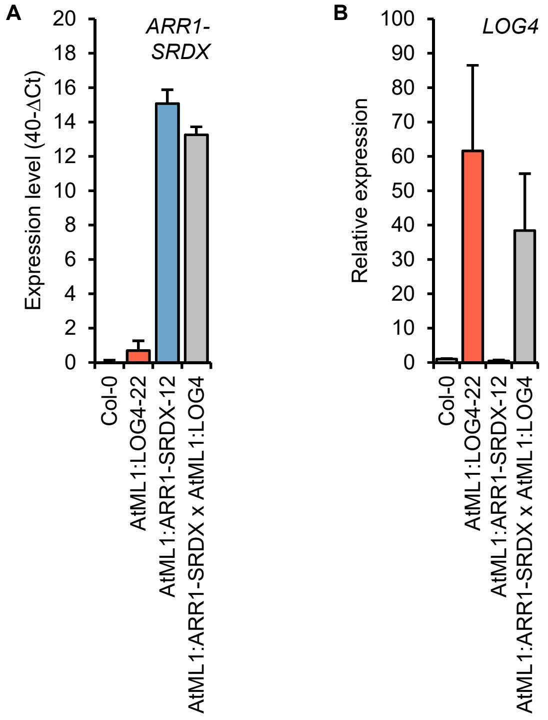

FIGURE S5 │ *AtML1:ARR1-SRDX* and *AtML1:LOG4* transgene expression in the hybrid transgenic lines. (A) Expression level of *ARR1-SRDX* and **(B)** *LOG4* in shoots of 10-d-old SD-grown plants. Transcript levels were determined by qRT-PCR, data were normalized to *TAFII15* (A) or *PP2AA2* and *TAFII15* (B), values are mean ± SEM, n = 3.
