# Supplementary material for: The Cytokinin Status of the Epidermis Regulates Aspects of Vegetative and Reproductive Development in Arabidopsis thaliana
Source: Front Plant Sci. 2021 Feb 23;12:613488. doi: 10.3389/fpls.2021.613488 (PMC7959818; doi:10.3389/fpls.2021.613488)
Supplement: Supplementary file 2 [file Data_Sheet_2.pdf]

SUPPLEMENTARY TABLES

TABLE S1 │ Cytokinin concentration in shoots of AtML1:LOG4 and AtML1:CKX1 plants.

	Col-0	AtML1:LOG4-17		AtML1:LOG4-22		AtML1:CKX1-32		AtML1:CKX1-37	
	Mean ± SD	Mean ± SD	% WT	Mean ± SD	% WT	Mean ± SD	% WT	Mean ± SD	% WT
iP	0.34 ± 0.07	0.39 ± 0.11	115	0.38 ± 0.04	112	0.23 ± 0.05	68	0.21 ± 0.07	62
iPR	1.82 ± 0.41	2.21 ± 0.69	121	1.62 ± 0.23	89	1.21 ± 0.09	66	**0.84 ± 0.12 ****	46
iPRMP	16.08 ± 2.13	13.52 ± 2.12	84	12.74 ± 1.57	79	**7.33 ± 1.01 ****	46	**8.26 ± 0.79 ***	51
iP7G	16.53 ± 1.67	17.14 ± 1.93	104	**24.04 ± 2.15 *****	145	**5.29 ± 0.58 *****	32	**5.72 ± 0.50 *****	35
iP9G	2.20 ± 0.30	**2.54 ± 0.13 ***	115	**3.07 ± 0.15 *****	140	**0.54 ± 0.05 *****	25	**0.57 ± 0.03 *****	26
*t*Z	1.35 ± 0.23	1.58 ± 0.18	117	**2.10 ± 0.05 *****	156	**0.42 ± 0.09 *****	31	**0.39 ± 0.02 *****	29
*t*ZR	2.01 ± 0.45	1.97 ± 0.28	98	1.95 ± 0.21	97	**0.33 ± 0.07 *****	16	**0.48 ± 0.11 *****	24
*t*ZRMP	17.73 ± 2.24	14.61 ± 2.55	82	15.32 ± 1.47	86	**3.98 ± 0.94 *****	22	**4.07 ± 0.66 *****	23
*t*Z7G	46.11 ± 5.94	**53.49 ± 4.62 ***	116	**59.51 ± 2.29 *****	129	**11.93 ± 1.54 *****	26	**13.13 ± 0.98 *****	28
*t*Z9G	14.99 ± 3.70	14.09 ± 1.65	94	16.83 ± 1.19	112	**3.28 ± 0.59 *****	22	**2.80 ± 0.62 *****	19
*t*ZOG	9.76 ± 1.15	**12.34 ± 0.85 ****	126	**15.10 ± 1.43 *****	155	**2.63 ± 0.48 *****	27	**3.48 ± 0.51 *****	36
*t*ZROG	0.49 ± 0.08	0.46 ± 0.08	94	0.46 ± 0.03	94	**0.11 ± 0.04 *****	22	**0.16 ± 0.02 *****	33
*c*Z	0.06 ± 0.01	**0.09 ± 0.01 ***	150	**0.12 ± 0.01 *****	200	0.05 ± 0.01	83	**0.03 ± 0.01 ****	50
*c*ZR	0.33 ± 0.06	0.31 ± 0.07	94	0.26 ± 0.03	79	**0.18 ± 0.01 ***	55	**0.17 ± 0.02 ****	52
*c*ZRMP	5.57 ± 0.96	4.81 ± 0.32	86	5.06 ± 0.35	91	**1.59 ± 0.24 ****	29	**1.85 ± 0.33 ***	33
*c*Z9G	0.09 ± 0.01	**0.11 ± 0.02 ***	122	**0.13 ± 0.01 *****	144	**0.02 ± 0.00 *****	22	**0.02 ± 0.00 *****	22
*c*ZOG	0.60 ± 0.21	0.55 ± 0.06	92	0.53 ± 0.03	88	0.55 ± 0.10	92	0.70 ± 0.19	117
*c*ZROG	0.70 ± 0.11	0.69 ± 0.07	99	0.66 ± 0.08	94	**0.52 ± 0.03 ****	74	**0.54 ± 0.02 ***	77

20 mg of shoot material of 7-d-old seedlings grown under LD conditions were harvested and pooled for every biological replicate. Values are pmol g FW ± SD (n = 4). Asterisks indicate significant differences compared to the wild type, as calculated by Kruskal-Wallis test, post-hoc Dunn‘s test (iP, iPRMP, *c*ZR, *c*ZRMP) or One-way ANOVA, post-hoc Dunnett‘s test (*, $p < 0.05$; **, $p < 0.01$; ***, $p < 0.001$). iP, N^6-isopentenyl adenine; iPR, iP riboside; iPRMP, iPR 5‘-monophosphate; iP7G, iP N^7-glucoside; iP9G iP N^9-glucoside; *t*Z, *trans*-zeatin; *t*ZR, tZ riboside; *t*ZRMP, *t*ZR 5‘-monophosphate; *t*Z7G, *t*Z N^7-glucoside; *t*Z9G, *t*Z N^9-glucoside; *t*ZOG, *t*Z *O*-glucoside; *t*ZROG, *t*ZR *O*-glucoside; *c*Z, *cis*-zeatin; *c*ZR, *c*Z riboside; *c*ZRMP, *c*ZR 5‘-monophosphate; *c*Z7G, *c*Z N^7-glucoside; *c*Z9G, *c*Z N^9-glucoside; *c*ZOG, *c*Z *O*-glucoside; *c*ZROG, *c*ZR *O*-glucoside.

TABLE S2 │ Mutants and transgenic lines used in this study.

Genotype	Named in this study	Reference
arr1-3 arr10-5	*arr1,10*	Argyros et al. (2008)
arr1-3 arr12-1	*arr1,12*	Mason et al. (2005)
arr10-5 arr12-1	*arr10,12*	Argyros et al. (2008)
ahk2-2tk cre1-12	*ahk2 cre1*	Higuchi et al. (2004)
ahk3-3 cre1-12	*ahk3 cre1*	Higuchi et al. (2004)
ahk2-2tk ahk3-3	*ahk2 ahk3*	Higuchi et al. (2004)

TABLE S3 │ Gene-specific primers used for genotyping in this study.

Locus	Allele	Primer pair	Sequences (5' → 3')	Fragment size
ARR1	WT	309_ARR1-arr1-3_fw<br>310_ARR1-arr1-3_rv	CTTCAAGCACTAGCCGTCACAGGTCAGTT<br>AATGTTATCGATGGAGTATGCGTCAAAGT	1306 bp
	arr1-3	309_ARR1-arr1-3_fw<br>315_LBa1	CTTCAAGCACTAGCCGTCACAGGTCAGTT<br>tggttcacgtagtgggccatcg	953 bp
ARR10	WT	311_ARR10-arr10-5_fw<br>312_ARR10-arr10-5_rv	CATTGGAGTTGTTGAGGGAGA<br>CGATGATGAGACTGGTTGGA	1075 bp
	arr10-5	311_ARR10-arr10-5_fw<br>315_LBa1	CATTGGAGTTGTTGAGGGAGA<br>tggttcacgtagtgggccatcg	1230 bp
ARR12	WT	313_ARR12-arr12-1_fw<br>314_ARR12-arr12-1_rv	TAACAACGACGAACCAAGCA<br>TTGGCAGAGTCACAGAATGG	1654 bp
	arr12-1	313_ARR12-arr12-1_fw<br>315_LBa1	TAACAACGACGAACCAAGCA<br>tggttcacgtagtgggccatcg	962 bp
AHK2	WT	446_AHK2-ahk2-2tk-2_fw<br>447_AHK2-ahk2-2tk-2_rv	TGCCTTGTCTATTCTTGTATCT<br>TAGGTTCAATTTCTTCAGTCC	719 bp
	ahk2-2tk	446_AHK2-ahk2-2tk-2_fw<br>230_T-DNA-ahk2-2tk_rv	TGCCTTGTCTATTCTTGTATCT<br>ataacgctgcggacatctac	~ 700 bp
AHK3	WT	397_AHK3-ahk3-3_fw<br>449_AHK3-ahk3-3_rv	GCAAGAATCCAGGTGCTAAC<br>GCTATCAGTTACAACCCTTGC	771 bp
	ahk3-3	315_LBa1<br>449_AHK3-ahk3-3_rv	tggttcacgtagtgggccatcg<br>GCTATCAGTTACAACCCTTGC	~ 850 bp
CRE1/AHK4	WT	213_CRE1-cre1-12_fw<br>214_CRE1-cre1-12_rv	ggagagccttcaccggttagg<br>aagctcttgcatttcatggaaatc	982 bp
	cre1-12	213_CRE1-cre1-12_fw<br>315_LBa1	ggagagccttcaccggttagg<br>tggttcacgtagtgggccatcg	~ 500 bp

TABLE S4 │ Primers used for cloning.

DNA fragment	Primer pair	Sequences (5' → 3')
pAtML1	076_pATML1-**attB1**_fw	**GGGGACAAGTTTGTACAAAAAAGCAGGCT**acaagccgtagatgattggttttcttctc
	077_pATML1-**attB2**_rv	**GGGGACCACTTTGTACAAGAAAGCTGGGTA**atagccggtcaagacataaccggtg
	078_pATML1-**attB4**_fw	**GGGGACAACTTTGTATAGAAAAGTTGTC**acaagccgtagatgattggttttcttctc
	079_pATML1-**attB1r**_rv	**GGGGACTGCTTTTTTGTACAAACTTGC**atagccggtcaagacataaccggtg
LOG4	168_LOG4-**attB1**_fw	**GGGGACAAGTTTGTACAAAAAAGCAGGCT**atggaggtcaacaatgaaaccatgc
	169_LOG4-**attB2**_rv	**GGGGACCACTTTGTACAAGAAAGCTGGGTA**tcagtcttcagaagagtagtcaatccgc
CKX1	030_CKX1-**attB1**_fw	**GGGGACAAGTTTGTACAAAAAAGCAGGCT**atgggattgacctcatccttacggttc
	059_CKX1-**attB2**_rv	**GGGGACCACTTTGTACAAGAAAGCTGGGTA**tacagttctaggtttcggcagtattgatgc
ARR1-SRDX	029_ARR1-**attB1**_fw	**GGGGACAAGTTTGTACAAAAAAGCAGGCT**atgatgaatccgagtcacggaagagg
	066_SRDX-**attB2**_rv	**GGGGACCACTTTGTACAAGAAAGCTGGGTA**agcgaaacccaaacggagttctagatc

TABLE S5 │ qRT primers used in this study.

Gene	Primers	Sequences (5' → 3')
PP2AA2	478_PP2AA2-qRT_fw	CCATTAGATCTTGTCTCTCTGCT
	479_PP2AA2-qRT_rv	GACAAAACCCGTACCGAG
TAFII15	339_TAFII15-qRT_fw	gaatcacggccaacaatc
	340_TAFII15-qRT_rv	actcttagccaagtagtgctcc
ARR4	490_ARR4-qRT_fw	CCGTTGACTATCTCGCCT
	491_ARR4-qRT_rv	CGACGTCAACACGTCATC
ARR5	593_ARR5-qRT_fw	CTACTCGCAGCTAAAACGC
	594_ARR5-qRT_rv	GCCGAAAGAATCAGGACA
ARR6	492_ARR6-qRT_fw	GAGCTCTCCGATGCAAAT
	493_ARR6-qRT_rv	GAAAAAGGCCATAGGGGT
ARR7	595_ARR7-qRT_fw	CTTGGAACCAATCTGCTCTC
	596_ARR7-qRT_rv	ATCATCGACGGCAAGAAC
ARR9	599_ARR9-qRT_fw	GATAGAGCACGTCCTAGATTCG
	600_ARR9-qRT_rv	CTGCATTCCCTACTGAAACC
LOG4	476_LOG4-qRT_fw	ATCAGTACAAACGCACGCCA
	477_LOG4-qRT_rv	GTCAATCCGCTCTATCTCCCA
CKX1	475_CKX1-qRT_fw	GGAAACAAGCCTACGACCCT
	476_CKX1-qRT_rv	GTTGCCTTTGACTTTGCGAG
ARR1-SRDX	472_ARR1-SRDX-qRT_fw	TGGCTACGGATACAGCAACAA
	473_ARR1-SRDX-qRT_rv	AGCGAAACCCAAACGGAGT

REFERENCES

Argyros, R.D., Mathews, D.E., Chiang, Y.H., Palmer, C.M., Thibault, D.M., Etheridge, N., Argyros, D.A., Mason, M.G., Kieber, J.J., and Schaller, G.E. (2008). Type B response regulators of *Arabidopsis* play key roles in cytokinin signaling and plant development. *Plant Cell* 20**,** 2102-2116.

Higuchi, M., Pischke, M.S., Mähönen, A.P., Miyawaki, K., Hashimoto, Y., Seki, M., Kobayashi, M., Shinozaki, K., Kato, T., Tabata, S., Helariutta, Y., Sussman, M.R., and Kakimoto, T. (2004). *In planta* functions of the *Arabidopsis* cytokinin receptor family. *PNAS* 101**,** 8821-8826.

Mason, M.G., Mathews, D.E., Argyros, D.A., Maxwell, B.B., Kieber, J.J., Alonso, J.M., Ecker, J.R., and Schaller, G.E. (2005). Multiple type-B response regulators mediate cytokinin signal transduction in *Arabidopsis*. *Plant Cell* 17**,** 3007-3018.
